# What Next?

**Published:** 1874-09

**Authors:** 


*PHILADELPHIA MEDICAL TIMES—August:*

What Next?—In a recent editorial we animadverted upon the methods of acquiring popular fame and practice which are becoming fashionable in New York. It may be that all our old notions of propriety are wrong, and that as the people are in reality to a great extent the arbiters in medical matters, so far at least as concerns *pecuniary* success, therefore it is better to spread before them all our knowledge and all our differences, so that a man may make up his judgment from the *Tribune*, or other of his daily papers, who is the best physician or surgeon of his neighborhood. Out of the attrition of the daily life of great cities usually come the sparks that light up revolutions; and where should a revolution start in this country if not in New York? Is the profession there ready for revolution?—ready to cast off the old garments of respectability, and to put on the new livery, whose color and fit are so close to that of the servants of quackery? We should judge not; yet time and again long articles appear in the *Tribune*, undoubtedly written by medical men, and undoubtedly written for the purpose of aiding those whose praises they sing. This is done, too, seemingly without a protest from any one; not a word against it in the New York medical press, not a whisper in the societies—at least that reaches the outside world.

The consultation room has been held the most sacred of all places; but in New York, when a man differs from his fellows, he, at least sometimes, goes for solace to the *Tribune*, and details in long, well-written articles the discussions of the consultation-room, the superiority of his own merits, and the grand improvements he has made in medical science; or, if he does not actually do this, he tells his subordinate all about the circumstances, and he does it for him.

The recent vagaries in regard to rabies are still fresh in the minds of men; but we have been incited to writing the present article by reading nearly two columns in the *Tribune* of July 14, detailing how Dr. William F. Fluhrer thought an arm could be saved when Dr. James R. Wood and others thought it could not, and how Dr. Fluhrer did save the arm, etc.

There are few things more pitiable than a man struggling vainly in a flood. We do not covet a martyr's destiny, and if the profession decide that the necessities of to-day require the appropriation of the quack's method by the regular profession, we have nothing to say; but when the spirit and letter of the Code of Ethics are opposed to such doings, it is but fair that all members of the profession in good standing should be forced to comply with this code.

